# Early Clinical Experience in the Management of Nodular Complications Following Cross-Linked Porcine Collagen Injection for Tear Trough Augmentation: A Two-Case Report

**DOI:** 10.7759/cureus.104457

**Published:** 2026-02-28

**Authors:** Edmond Lau, Stephanie Y Ming, Kyuho Yi

**Affiliations:** 1 Aesthetics, EC Skin Laser Aesthetic Clinic, Penang, MYS; 2 Oculoplastic Surgery, Eagle Eye Centre, Singapore, SGP; 3 Anatomy, Yonsei University, Seoul, KOR

**Keywords:** filler complication, filler complication management, nodular complication, porcine collagen filler, tear trough filler, tear trough rejuvenation, ultrasound-guided

## Abstract

Cross-linked porcine collagen fillers have been utilized for tear trough augmentation, primarily for the correction of dark under-eye circles. However, when nodular complications occur, their management remains challenging due to the absence of predictable enzymatic degradation and the lack of standardized treatment protocols.

Two patients developed non-inflammatory infraorbital nodules following tear trough augmentation with cross-linked porcine collagen filler. Ultrasound examination demonstrated well-defined hypoechoic lesions within the superficial subcutaneous plane. Both cases were treated with ultrasound-guided intralesional injection using sterile water for injection (SWFI) combined with lidocaine, with hyaluronidase added in one case, followed by local massage. Complete clinical and ultrasonographic resolution was observed within one day and one week, respectively.

Based on these two cases, ultrasound-guided intralesional management using SWFI facilitates the mechanical dispersion of aggregated collagen filler and represents a safe, minimally invasive approach for treating nodular complications in the tear trough. Early ultrasound assessment enables accurate diagnosis and targeted intervention in this anatomically delicate region.

## Introduction

Tear trough deformity is an aesthetic concern frequently addressed through nonsurgical augmentation. Injectable soft tissue fillers have become a mainstay treatment due to their minimally invasive nature, high patient satisfaction, and relatively short recovery period [1]. While hyaluronic acid (HA) fillers remain the most commonly used material, recent publications from Asia have described the application of cross-linked porcine collagen injections in the periorbital region to treat structural dark undereye circles and tear trough [2,3].

The infraorbital region presents unique challenges for filler injection due to its thin skin. This anatomical characteristic predisposes patients to issues such as the Tyndall effect and persistent nodularity or edema [4,5]. While HA fillers remain the most widely used material for tear trough augmentation, nodule formation has been reported as a rare but recognized complication, with incidence estimates ranging from approximately 1% to 4%, depending on product type and injection site [1].

While collagen fillers do not typically cause the Tyndall effect, the risk of nodule formation from excessive volume injected too superficially can still occur [6]. Despite this, the literature on newer, cross-linked porcine collagen fillers is limited, and reliable incidence data for nodule formation are lacking. Studies on porcine collagen have reported widely variable rates of nodules and papules, depending on the product, injection technique, and treatment site. For example, one study evaluating the application of cross-linked porcine collagen filler Therafill® (K Derma Co. Ltd, South Korea) in the nasolabial fold area reported no cases of nodules among 57 subjects [6]. In contrast, another study using Evolence® (Ortho Neutrogena, Los Angeles, CA) for lip augmentation reported nodular formation in 80% of patients [7]. Studies utilizing Deusaderm Lido (Sunmax Biotechnology Co. Ltd., Taiwan) over the undereye region have been shown to be safe, and no nodular complication was described [2,3].

While HA fillers are degradable with hyaluronidase and thus complications can be corrected [8-10], dealing with complications secondary to collagen-based injectables, especially the cross-linked variants, may require an alternative strategy. However, there remains a limited published consensus or standardized management protocol for nodular complications arising from cross-linked collagen products injected into the under-eye region.

In this report, we present two cases of nodular complications after lower eyelid and tear trough augmentation with cross-linked porcine collagen filler (Deusaderm Lido). We describe the clinical presentation, ultrasound assessment, and treatment protocol, aiming to contribute a practical management framework for similar adverse events in aesthetic practice.

## Case presentation

Case 1

**Figure 1:**
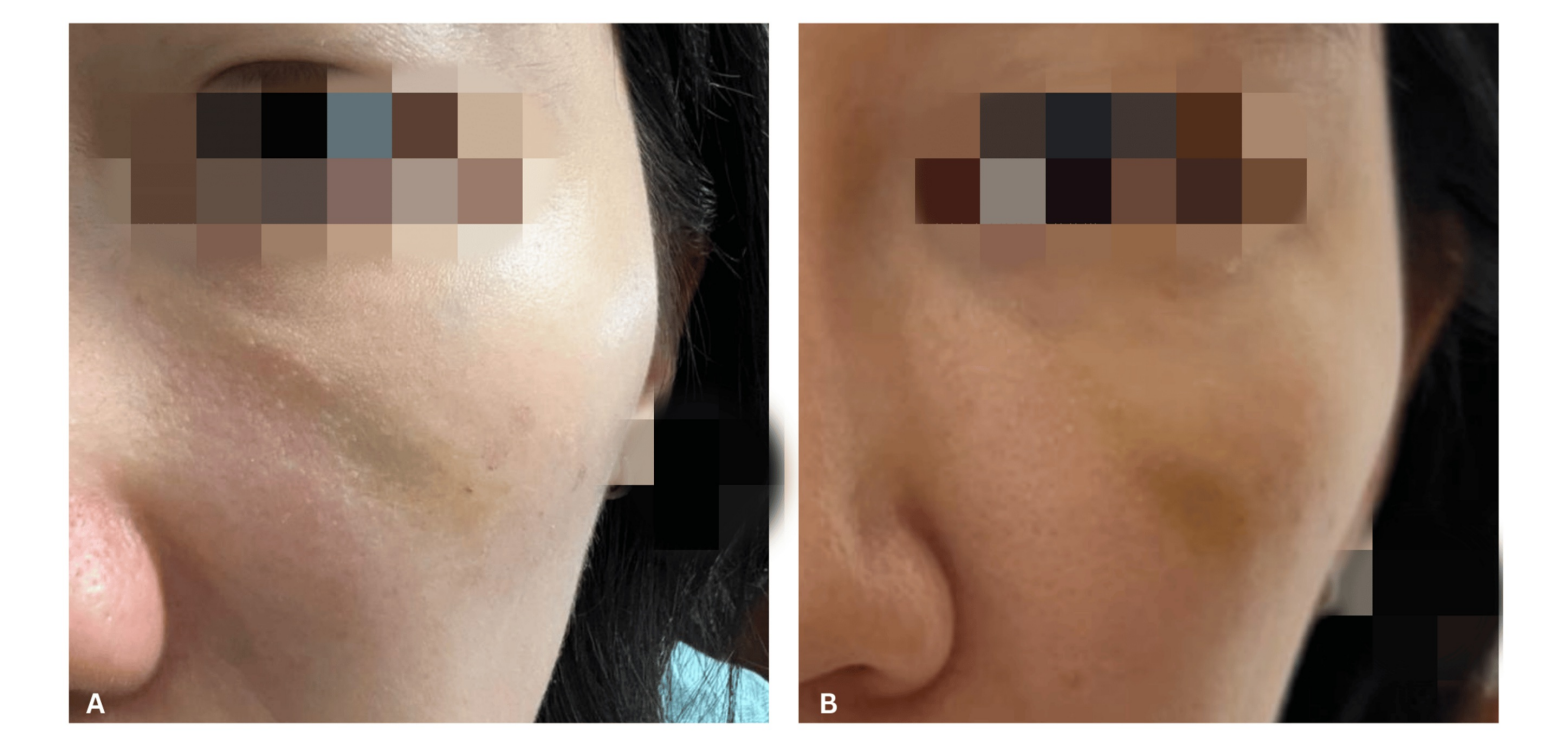
Clinical appearance of the tear trough nodular complication before and after ultrasound-guided treatment (Case 1) (A) Clinical photograph demonstrating a palpable nodule over the left tear trough region prior to treatment. (B) Clinical photograph of the left tear trough region one day post-treatment, showing marked resolution of the nodule.

**Figure 2:**
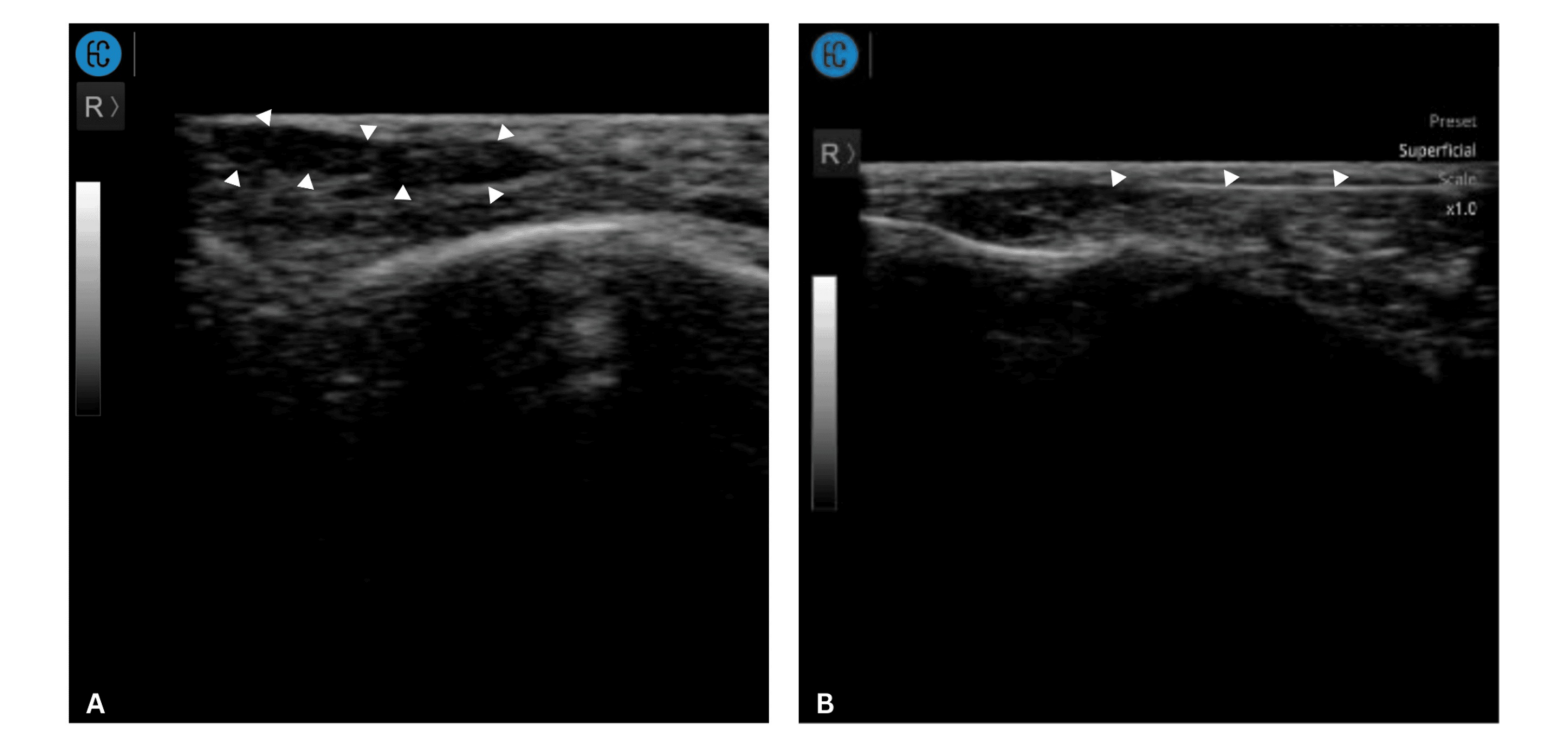
Ultrasonographic findings and ultrasound-guided treatment of the tear trough filler nodule (Case 1) (A) Ultrasound image of the left infraorbital region in the oblique plane demonstrating a well-defined hypoechoic lesion (indicated by arrowheads) within the superficial subcutaneous layer, located superficial to the orbicularis oculi muscle, consistent with aggregated filler material. (B) Ultrasound image obtained during ultrasound-guided intralesional injection of the filler nodule with arrowheads indicating the cannula.

A 36-year-old woman presented to the clinic with a non-inflamed nodule over her left tear trough after she had cross-linked porcine collagen filler injected 10 days prior (Figure 1A). Point-of-care ultrasound (POCUS) examination (Sonon 500L, 12MHz, Linear Probe, Healcerion, South Korea) over the nodule noted a distinct subdermal hypoechoic lesion (Figure 2A). Under ultrasound guidance, an intralesional injection consisting of 0.8 mL of sterile water for injection (SWFI), mixed with 0.2 mL of 2% lidocaine, was administered using a 25-gauge, 50 mm cannula. The in-plane ultrasound-guided technique was used throughout the procedure to confirm that the cannula tip remained within the area of product aggregation (Figure 2B). The cannula entry point was located over the malar eminence, and the injection was performed over three to four passes. Local massage was performed post-injection. During follow-up the next day, the nodule had fully resolved (Figure 1B). At the one-month review, the resolution was maintained with no recurrence.

Case 2

**Figure 3:**
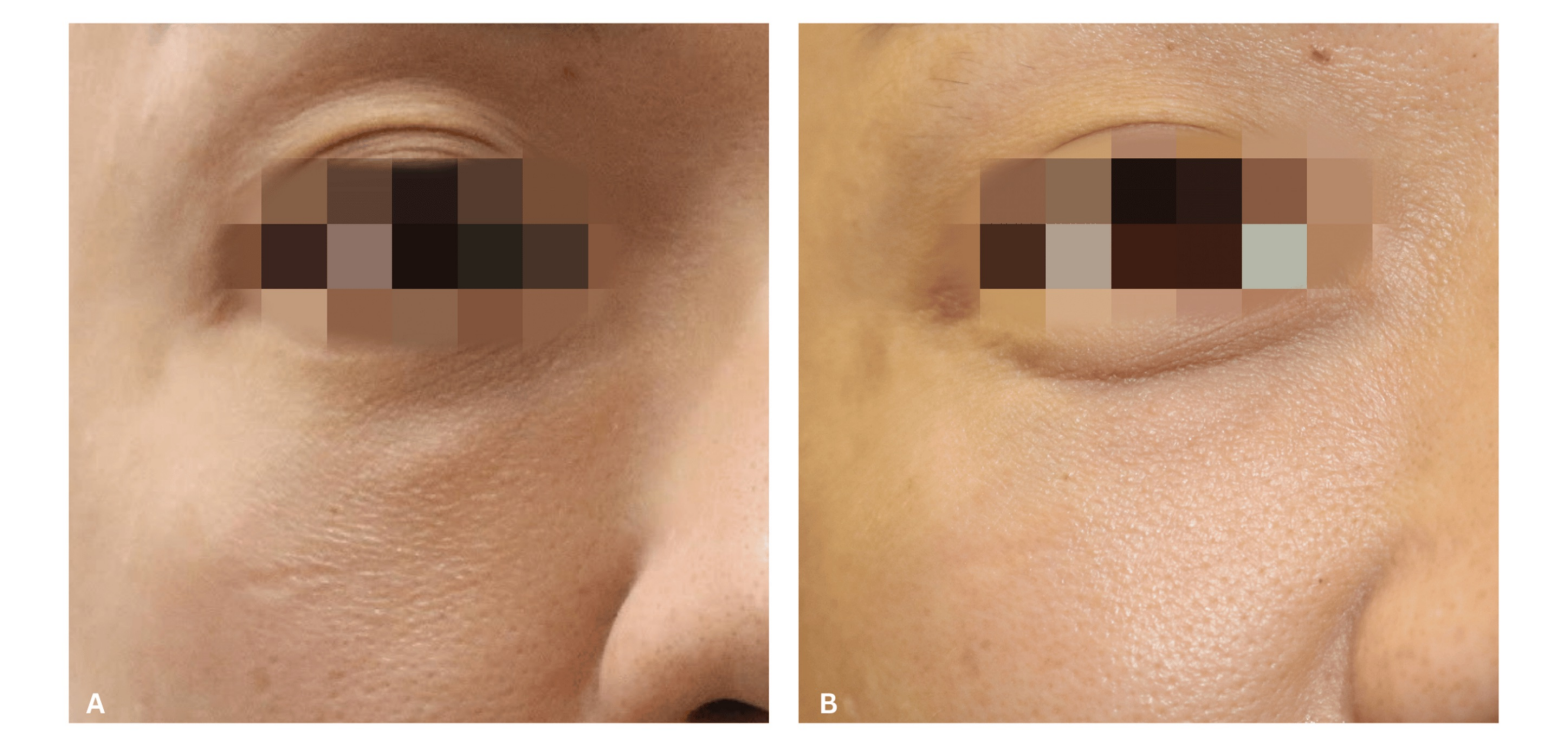
Clinical appearance of infraorbital nodular complication before and after ultrasound-guided treatment (Case 2) (A) Clinical photograph demonstrating a palpable nodule over the right infraorbital region prior to treatment (B) Clinical photograph of the right infraorbital region one week post-treatment, showing marked resolution of the nodule.

**Figure 4:**
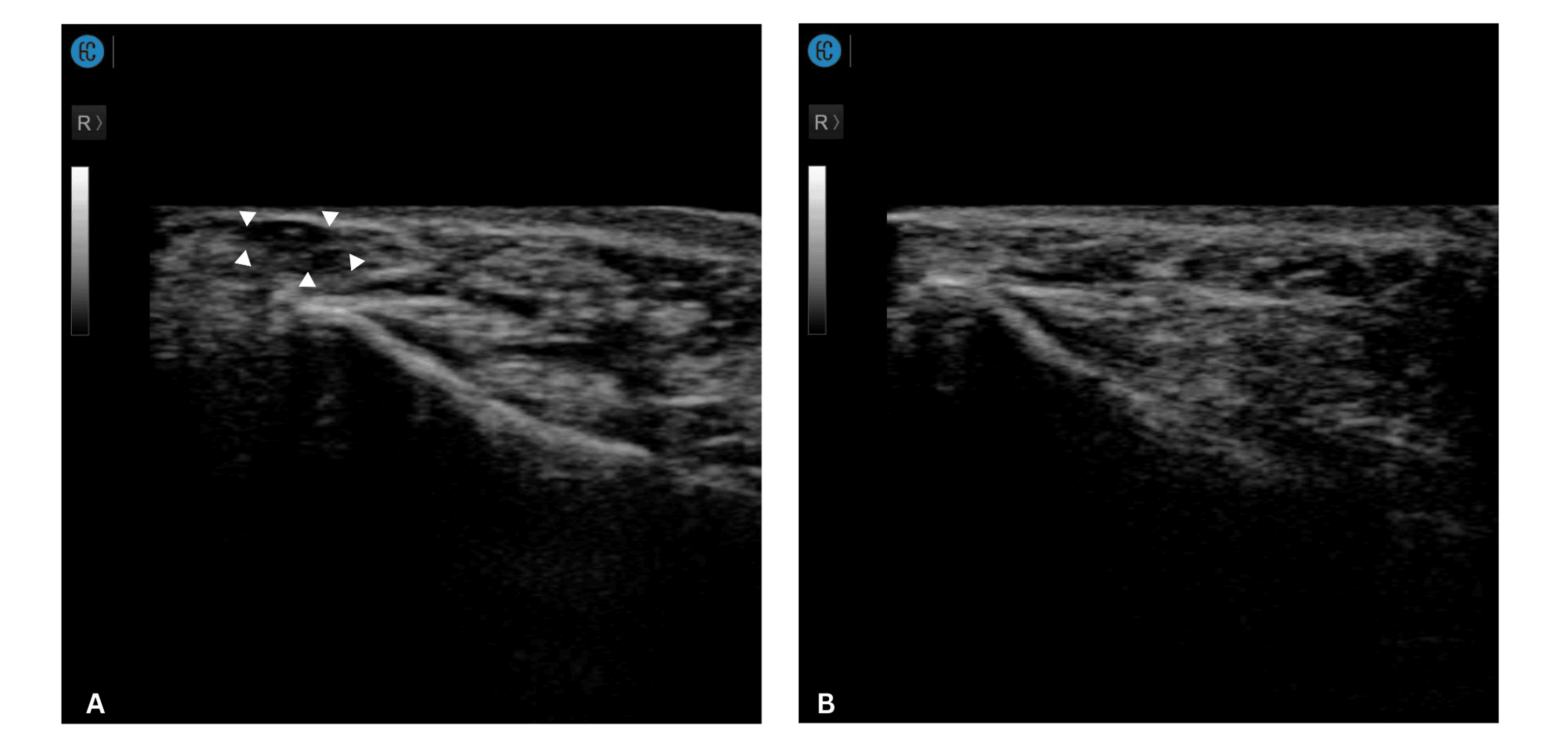
Ultrasonographic appearance of the tear trough filler nodule before and after treatment (Case 2) (A) Ultrasound image of the right infraorbital region in the oblique plane demonstrating a well-defined hypoechoic lesion (indicated by arrows) within the superficial subcutaneous layer, consistent with aggregated filler material. (B) Follow-up ultrasound image of the right infraorbital region obtained one week post-treatment, showing complete resolution of the previously observed hypoechoic lesion.

A 35-year-old man presented to the clinic with a single, visible, non-inflamed nodule over the right lower eyelid that occurred after he had cross-linked porcine collagen filler injected under the eye 2 months prior (Figure 3A). Previous attempts to dissolve the nodule using hyaluronidase with normal saline (NS) yielded no results. POCUS examination (Sonon 500L, 12MHz, Linear Probe) noted a distinct hypoechoic lesion located superficial to the orbicularis oculi muscle (Figure 4A). A solution of 100 iu hyaluronidase diluted in 1 ml of SWFI plus 0.2 mL of 2% lidocaine was injected into the nodule under ultrasound guidance, followed by local massage. During follow-up at one week, the nodule had resolved (Figure 3B, Figure 4B). Subsequent evaluation at one month confirmed sustained resolution with no evidence of recurrence.

## Discussion

Nodular complications following tear trough augmentation remain a challenging adverse event, particularly when non-hyaluronic acid fillers are involved. The infraorbital region is uniquely vulnerable due to the thin dermis, minimal subcutaneous fat, and high aesthetic visibility. Even minor irregularities or volume misplacement can result in clinically apparent nodules, which are often distressing to patients and difficult to manage [4,8].

Cross-linked porcine collagen fillers have gained popularity in Asia for periorbital rejuvenation, especially in patients presenting with dark under-eye circles [2,3]. Compared with HA fillers, collagen-based products are less likely to produce the Tyndall effect due to their optical properties [11]. However, the bioproperty of collagen filler and cross-linked structure also means that complications cannot be addressed using conventional enzymatic degradation with hyaluronidase in a predictable manner. This creates a therapeutic gap when nodules or irregularities arise [12,13].

The cases presented in this report illustrate that nodular complications following tear trough augmentation with cross-linked collagen filler may arise when relatively larger volumes of product are deposited within the superficial tissue plane. Both patients exhibited non-inflamed nodules, indicating product aggregation rather than an inflammatory etiology. Ultrasound examination noted well-defined hypoechoic lesions localizing the nodule to the superficial subdermal plane, above the orbicularis oculi muscle. Ultrasound imaging played a pivotal role in both the diagnosis and management of our cases. POCUS allowed precise localization of the filler material, confirmation of its depth, while ultrasound-guided intralesional injection ensured accuracy of needle placement.

Interestingly, both cases responded well to intralesional dilution using SWFI combined with lidocaine, with or without hyaluronidase. In Case 1, complete resolution was achieved using SWFI and lidocaine alone, suggesting that mechanical dilution and redistribution of superficially placed collagen may be sufficient in early nodules. In Case 2, hyaluronidase was included despite the filler being collagen-based. While hyaluronidase does not enzymatically degrade collagen, its use may have contributed indirectly through increased tissue permeability and facilitation of dispersion when combined with fluid infiltration and massage [14].

Although direct studies comparing SWFI and NS for the dispersion of filler aggregation are lacking, the use of hypotonic and isotonic solutions may influence biological effects beyond simple volume expansion. The rationale for using SWFI rather than NS lies in the isotonic nature of saline, which does not induce significant osmotic shifts across tissue compartments and may therefore limit its ability to disperse aggregated filler material. In contrast, SWFI is hypotonic and can generate transient osmotic gradients that promote mechanical fluid movement within tissue planes [15].

While no direct clinical trials have evaluated NS versus SWFI in the management of skin nodules from cross-linked porcine collagen filler, experimental work by McCarthy et al. demonstrated that sterile water injected into calcium hydroxylapatite nodules, followed by vigorous massage, resulted in greater dispersion compared with NS plus massage or massage alone [16]. Furthermore, Chen et al. showed that non-ionic hypotonic diluents like SWFI improved the solubility of carboxymethyl cellulose (CMC) gel, thereby enhancing the dispersion and homogeneity of poly-d,l-lactic acid particles compared to NS, supporting the theoretical advantage of hypotonic fluids in facilitating mechanical dispersion [17].

At present, there is no standardized protocol for managing non-inflammatory nodule complications from cross-linked porcine collagen fillers in the tear trough [8]. Based on these cases, a preliminary clinical approach can be suggested. Early ultrasound assessment is recommended to confirm the diagnosis and determine the depth of the nodule, followed by ultrasound-guided intralesional dispersion using SWFI with or without adjunctive agents, such as hyaluronidase or lidocaine, combined with massage. Collagenase may be considered for refractory nodular complications arising from collagen; however, its use is limited by restricted availability, lack of standardized treatment protocols, and the risk of non-selective degradation of native collagen [12,13].

This report has several important limitations. First, the sample size is extremely small, consisting of only two cases, and there is no control group for comparison. The follow-up duration was short, limiting the ability to assess long-term outcomes or recurrence. The treatment approach described is operator-dependent and may not be reproducible across different practitioners. Additionally, the intervention may involve off-label use, and objective volumetric measurements were not performed. Spontaneous resolution of the nodules cannot be fully excluded as a contributing factor. Finally, the absence of histologic confirmation limits the ability to definitively characterize the nature of the nodules. It remains unclear whether the disruption of the nodule was caused by the needle puncture itself, the instillation of sterile solution, or the subsequent massage applied to the area, making it difficult to identify the primary mechanism of effect.

## Conclusions

In conclusion, while cross-linked collagen fillers offer aesthetic advantages in the treatment of tear trough deformity, they are not without risk, and nodular complications can occur. Based on these two cases, ultrasound-guided intralesional management using SWFI facilitates mechanical dispersion of aggregated collagen filler and represents a safe, minimally invasive approach for treating nodular complications in the tear trough. Early ultrasound assessment enables accurate diagnosis and targeted intervention in this anatomically delicate region.

Further studies with larger case series are needed to establish standardized guidelines and clarify the role of adjunctive agents, such as hyaluronidase and collagenase, in managing complications associated with non-hyaluronic acid fillers. Additionally, controlled investigations that isolate each intervention, such as needle puncture, SWFI instillation, and massage, would help determine their individual contributions to nodule resolution.
